# In the Light of Healthcare Professionals: Beliefs About Chronic Low Back Pain

**DOI:** 10.3390/medicina62010183

**Published:** 2026-01-16

**Authors:** Brigitta Péter, Adrian Georgescu, Ileana-Monica Popovici, Lucian Popescu, Timea Szabó-Csifó, Liliana-Elisabeta Radu, Pia-Simona Fagaras

**Affiliations:** 1 SC Procardia Health SRL, 540043 Târgu Mureș, Romania; brigittapeter@gmail.com; 2 Faculty of Physical Education and Sport, “Ovidius” University, 900527 Constanța, Romania; adrian_georgescu_78@yahoo.com; 3 Faculty of Physical Education and Sport, “Alexandru Ioan Cuza” University of Iasi, 700506 Iași, Romania; ileana.popovici@uaic.ro (I.-M.P.); lucian_popescu2009@yahoo.com (L.P.); 4 County Emergency Clinical Hospital, 540136 Târgu Mures, Romania; dr.szabocsifo@gmail.com; 5 Motor Science Discipline, Department M2, “G.E. Palade” University of Medicine, Pharmacy, Science and Technology, 540142 Târgu Mures, Romania

**Keywords:** low back pain, healthcare, beliefs, chronic pain, pain knowledge

## Abstract

*Background and Objectives:* Chronic low back pain (CLBP) is a prevalent condition that impairs quality of life, functionality, and work productivity. While most acute episodes of back pain resolve, 4–25% become chronic due to factors such as high pain intensity, psychological distress, and maladaptive behaviors. Nonspecific CLBP is best understood through the biopsychosocial model, encompassing biological, psychological, and social influences, including kinesiophobia. Management relies on physical activity, pain education, and psychological interventions, with therapist knowledge and attitudes affecting outcomes. This study aimed to assess the prevalence of CLBP among healthcare workers, examine their knowledge of pain neurophysiology, evaluate kinesiophobia, and explore how personal experience with CLBP influences their beliefs, attitudes, and interactions with patients. *Materials and Methods:* A cross-sectional observational study was conducted from January to May 2025 among healthcare professionals. A total of 50 participants completed an online questionnaire, of which 42 were valid and included in the analysis. The questionnaire collected demographic and professional data, determined the presence of CLBP, and included three standardized instruments: the Revised Neurophysiology of Pain Questionnaire (rNPQ) to assess knowledge of pain mechanisms, the Health Care Providers’ Pain and Impairment Relationship Scale (HC-PAIRS) to evaluate beliefs about pain and disability, and the Tampa Scale of Kinesiophobia (TSK-11) to measure fear of movement. Data were analyzed using SPSS and Microsoft Excel. *Results:* Among the 42 participants, 11 demonstrated low, 28 moderate, and 3 high knowledge of pain neurophysiology (rNPQ), with a mean score of 5.66. On the HC-PAIRS, the majority (30 participants) scored above 60, indicating beliefs that pain leads to disability, while 12 scored below 60, reflecting a biopsychosocial perspective; gender did not significantly affect HC-PAIRS scores (*p* = 0.213). As for kinesiophobia (TSK-11), 24 participants had low, 17 moderate, and 1 clinically significant fear of movement. Correlation analysis revealed that younger participants had higher rNPQ scores (r = −0.358, *p* = 0.020) and lower TSK-11 scores (r = −0.389, *p* = 0.011). TSK-11 scores increased with age (r = 0.432, *p* = 0.004), while HC-PAIRS scores showed no significant correlations. *Conclusions:* Healthcare professionals, particularly physiotherapists, show gaps in knowledge of pain neurophysiology and a tendency toward biomedical beliefs regarding chronic low back pain. This cross-sectional study indicates that a greater understanding of pain mechanisms is associated with lower kinesiophobia, emphasizing the importance of education. Integrating the biopsychosocial model into undergraduate and continuing professional training, through interdisciplinary and practical modules, may improve knowledge, reduce maladaptive fear-avoidance behaviors, and enhance patient care. Future studies should include larger, more diverse samples and assess the long-term impact of educational interventions on clinical practice.

## 1. Introduction

Chronic low back pain (CLPB) is one of the most frequently encountered musculo-skeletal conditions worldwide, having a significant impact on the patient’s quality of life and functional capacity. CLBP is the leading cause of years lived in disability in developed countries; it has gained increasing importance not only from a medical perspective but also from a socioeconomic one by generating substantial costs associated with absenteeism, reduced productivity, and healthcare services [1]. The prevalence increases among individuals over the age of 40 and among women [2].

The diagnostic process starts with a detailed medical history and physical examination. Based on the doctor’s decision, the examination can be followed by an imaging evaluation, either an X-ray or a magnetic resonance imaging, to define the cause of the LBP [3], though evidence suggests that if there are no “red flags” in the medical history, imaging is not recommended in the first 6 weeks because it may worsen the anxiety of the patient [4]. “Red flags” are signs that suggest a severe underlying cause that needs immediate medical attention, such as age below 18 years, fever, recent spinal surgery or injection, immunosuppressant medication, etc. [5].

In most cases, the acute symptoms improve within one month, however 4–25% of cases become chronic. Prognostic factors for chronicity are higher intensity of pain, functional limitation, high physical demands at work, maladaptive behavior strategies, smoking, obesity, anxiety, a high level of emotional distress, and depression [6,7].

Low back pain can be categorized by cause as either specific or a nonspecific [8]. Specific LBP has an identifiable cause of spinal origin (ex. sciatica, spinal stenosis, disk herniation) or nonspinal origin (ex. endometriosis, coxarthrosis). On the other hand, the cause nonspecific LBP cannot be identified: it most likely develops out of a combination of biologic, social, and psychological factors. In current medical practice, this condition is regarded more as a neurobiological and behavioral response to an individual’s perception of danger than as a disease in itself [9].

Thus, the biopsychosocial model became extremely relevant in the context of nonspecific LBP. Formulated by George L. Engel in 1977, the biopsychosocial model states that an illness is a result of a complex interaction between different factors, and cannot be explained exclusively by physiopathological processes [10]. Psychological factors, such as anxiety, depression, pain catastrophizing, and fear-avoidance beliefs contribute to maintaining and increasing the pain, as well as limiting the patients’ functional abilities. Social factors, for example, family and occupational support, work conditions, and socio-economic status, also make a difference in the evolution of pain and pain management [11]. Biological factors include tissue-modification in the lower back area, sensibilization of the nervous system (hyperalgesia, allodynia), motor control dysfunction, genetics, and comorbidities [12].

Another important psychological aspect of treating CLBP is kinesiophobia, the fear of movement that can lead to avoidance of physical activity. Kinesiophobia is strongly related to pain intensity, level of disability, and quality of life of CLBP patients, which suggests that it can contribute to pain aggravation and functional disability [13]. The treatment includes physical approaches (physical therapy, exercising) [14], psychological approaches (cognitive behavior therapy, mindfulness-based therapy) [15], and pain education [16].

Prevention and treatment of LBP is challenging in a high-risk population. Physical activity is considered essential, and combined with education it has better outcomes [17]. Compared with other noninvasive methods, physical activity is more effective in reducing pain and increasing functionality [18]. Apart from exercise, the current European guidelines recommend the following: patient reassurance, offering detailed advice and education about physical activity and work, manual therapy, cognitive compartmental therapy, and professional reintegration programs. Surgical intervention is advised only in cases where noninvasive treatment failed, there is severe persistent pain, or if there is a severe underlying condition (ex. Cauda equina syndrome or severe neurological deficits) [19,20]. Pain education has multiple benefits, such as reducing fear and anxiety by understanding the mechanism of pain; reducing fear-avoidance of physical activity; increasing self-efficiency, and modifying the perception of pain. Pain education can also affect behavior, increasing adherence to exercise therapy [21,22].

Another interesting aspect of the rehabilitation process may be the therapist’s attitude, which greatly influences the therapeutic relationship, and consequently the effectiveness of treatment. Therapists who are more biopsychosocially oriented and contemplate pain as a complex phenomenon have better treatment outcomes of CLBP, compared with those who are more biomedically oriented [23]. The therapist’s personal experience in managing pain can enhance empathy, provide deeper emotional support, and increase the patient’s confidence in the therapeutic process [24]. Understanding the neurophysiology of chronic pain allows the therapist to explain the mechanisms of pain to the patient and reduce the fear associated with it by helping the patient understand the pathology and providing reassurance. This therapeutic intervention requires professional training in biopsychosocial research and periodic updating of knowledge by staying informed through new research [25].

The aim of this study was to assess the prevalence of CLBP in healthcare workers, and to evaluate the extent to which kinesiophobia occurs in this studied group; to analyze the quality of their knowledge related to the neurophysiology of pain; and to examine the beliefs and attitudes of healthcare professionals regarding chronic low back pain.

## 2. Materials and Methods

### 2.1. Study Design

A cross-sectional observational study was conducted in January through to May of 2025. The research was conducted between January and March 2025 and involved administering questionnaires to assess the prevalence of chronic low back pain among healthcare workers, examine their understanding of pain neurophysiology, and evaluate kinesiophobia. Data collection was performed through online questionnaires using Google Forms, producer Google LLC (Mountain View, CA, USA) which ensured convenience and accessibility. Participation in the questionnaire was voluntary and adhered to ethical standards. The selection of the questionnaires was guided by recommendations from expert sports therapists and a comprehensive review of the relevant literature. Informed consent was obtained from each participant before data collection, and participation remained voluntary throughout the study. This research adhered to the principles of the Declaration of Helsinki and received approval from the Ethics Committee of “G.E. Palade” University of Medicine, Pharmacy, Science, and Technology from Targu Mures (protocol 527/10.12.2024).

### 2.2. Participants

The study involved 50 individuals of both genders, aged between 24 and 43 years. The inclusion criteria were that the participants worked in the healthcare field and correctly completed the form. A total of 42 participants were validated, the rest were excluded due to incomplete answers.

### 2.3. Assessment Tool

The form was divided into 2 sections: the first included questions about personal information, and the second part comprised three standardized questionnaires: the Revised Neurophysiology of Pain Questionnaire (rNPQ), the Health Care Providers’ Pain and Impairment Relationship Scale (HC-PAIRS), and the Tampa Scale of Kinesiophobia (TSK-11). After collecting the data, the responses were centralized and evaluated. Statistical analysis was conducted by the IBM Statistical Package for the Social Sciences (SSPS) version 20.0 and Microsoft Excel program (part of Microsoft Office, version 2010).

The rNPQ is a frequently used method for assessing an individual’s level of understanding of the biological mechanisms underlying their pain [26] and for evaluating the effects of clinical pain education programs [27]. The questionnaire consists of 13 items, possible responses are True, False, and Undecided, and every correct answer is worth a point. Higher scores indicate a better understanding of the neurophysiology of pain. The interpretation used in this study is that 0–4 points mean low quality of pain knowledge, 5–8 moderate, and above 9 means a profound understanding of the neurophysiology of pain.

The HC-PAIRS measures health care workers’ beliefs about the relationship between a patient’s pain and the level of their impairment. It has 15 items, graded on a 7-point Likert scale. A higher score (above 60), indicates stronger beliefs that pain inevitably leads to disability, whereas a lower score (below 60) reflects beliefs aligned with the biopsychosocial model, which states that chronic pain is not necessarily linked to disability [28].

The TSK-11 autoevaluates kinesiophobia (fear of movement) of patients who suffer from chronic pain caused by different musculo-skeletal pathologies [29], and is based on the fear-avoidance model: pain-related fear can lead to avoiding physical activity, which can intensify or sustain the pain [30]. The revised, 11-item scale is the most valid and reliable form of the TSK. Each items is graded on a 4-point Likert scale. Scores between 11 and 22 points are considered as low or non-existent kinesiophobia, between 23 and 33 a moderate level of kinesiophobia, and above 34 reflects a clinically significant fear of movement [31].

## 3. Results

The summarized results for the participants group are presented in Table 1. The distribution provides insights into the demographic and professional background of study participants, as well as their personal experiences with chronic low back pain.

### 3.1. Results of the rNPQ

Based on the statistical analysis, it can be observed that 11 participants obtained scores that indicate a low level of pain knowledge; 28 demonstrate a moderate level of knowledge; and only 3 participants have a high level of knowledge about the neurophysiology of pain. Table 2 presents the descriptive statistics for the rNPQ across the different interpretation levels and age groups.

### 3.2. Results of the HC-PAIRS

Based on the data analysis of the HC-PAIRS questionnaire, the maximum score was 92 out of a possible 105, and the minimum was 23 out of 105. Table 3 presents the distribution of the scores separated out by gender and age.

The <25 age group had the most participants with scores above 60 (15), indicating a potential bias in pain perception and impairment awareness within this age group. These results indicate a significant disparity in HC-PAIRS scores, both by gender and age. The majority of participants reported a better understanding of the relationship between pain and impairment, particularly among women and those aged <25, suggesting specific areas for future training and education.

To see if there is a statistically significant difference between males and females regarding the HC-PAIRS score, independent sample *t*-tests were conducted, accompanied by Levene’s Test for Equality of Variances. (Table 4). The result (*p* = 0.213) indicates that gender does not significantly influence the perceptions about chronic pain. These findings suggest that gender does not play a significant role in the perception of pain and impairment among healthcare providers.

### 3.3. Results of the TSK-11

The maximum obtained score on the TSK-11 was 35 points (clinically significant kinesiophobia), and the minimum scale was 11 points (non-existent kinesiophobia). Table 5 presents the descriptive analysis of the TSK-11 scores based on gender and age. The majority of the participants under the age of 30 registered scores that show minor kinesiophobia. These results reveal distinct patterns of kinesiophobia scores, both by gender and age. Women showed a higher incidence of low to moderate levels of kinesiophobia compared to men. In addition, the younger age group (<25) showed a notable trend towards lower kinesiophobia, while older participants (>40) showed a mixed range of scores, including one high score.

### 3.4. Correlation Between the Scales

Most importantly, we aimed to examine whether there are correlations between the scales. Table 6 presents the data for the statistical analysis.

Based on the analysis of the correlation between the knowledge of pain neurophysiology (rNPQ), beliefs about the relationship between chronic pain and disability (HC-PAIRS), and fear of movement (TSK-11) across four different age groups, we can state the following:

There was a significant negative correlation between rNPQ scores and age (r = −0.358, *p* = 0.020), suggesting that younger individuals are more informed on the mechanisms of pain.

Regarding the HC-PAIRS scores, no significant correlation was observed with any of the other variables: TSK scores (r = 0.214, *p* = 0.173) or age (r = −0.241, *p* = 0.124). This indicates that the beliefs of chronic pain measures by the HC-PAIRS are either relatively stable or influenced by other factors not covered by this study.

Concerning the TSK score, a significant positive correlation with age was observed (r = 0.432, *p* = 0.004), as kinesiophobia is more pronounced in older individuals. Statistical analysis revealed a significant negative correlation between the scores obtained on the rNPQ and TSK scores (r = −0.389, *p* = 0.011), which indicates that a higher level of knowledge about pain neurophysiology is associated with lower level of kinesiophobia, confirming that pain education can reduce fear-avoidance behavior.

## 4. Discussion

Results of the statistical analysis highlight several aspects of healthcare workers’ knowledge and beliefs toward CLBP, as well as demographic factors that may influence these perspectives. International research consistently reports a high incidence of CLBP among healthcare workers, as LBP is one of the most common issues among this occupational group [32], physical therapists and nurses being the most vulnerable due to manual patient handling and prolonged standing [33].

Our studied group shows an unusually high prevalence of CLPB of over 80%. Similar results were obtained by studies conducted in Saudi Arabia (74.2%) and China (72.8%) [34,35]. Other studies show a much lower, but significant, prevalence of around 57–59% in Ethiopia, Pakistan, Tunisia, and South Africa [33,36,37,38]. The globally high prevalence of CLBP emphasizes the relevance of the subject, with the need to explore different aspects of chronic pain.

The predominance of physiotherapists in this study is evident, as this occupational group accounts for more than half of the respondents (57.14%). This reflects the increased interest of this professional category in the subject, as they interact directly with CLBP patients. However, this unequal distribution of the participants based on occupation limits the potential for extrapolation of the conclusions to other healthcare professions.

The results obtained from administering the rNPQ provide a detailed overview of healthcare professionals’ knowledge of pain neurophysiology, showing that the majority of the participants understand the biological mechanisms of pain on a moderate level. Nevertheless, a significant proportion of participants display deficiencies in the comprehension of this subject. Accordingly, 26.19% of the participants scored at a low level of pain knowledge, 66.66% obtained a score suggesting moderate level, and only 7.14% received high scores. Our results align with the findings of a study conducted among physical therapists in Saudi Arabia, which reported an average score of 6.7, with 90% of the participants scoring 9 or lower [39].

As for the mean percent of correct responses, our study sample obtained 43.53%, which is below international standards. A study evaluating Spanish healthcare workers reported 67% correct answers. Similarly, a German article evaluating physical therapy students reported 75% [40,41]. This comparison highlights the importance of continuous professional development, aimed at updating scientific knowledge and aligning with modern, evidence-based practices.

The observed negative correlation between the level of knowledge of pain neurophysiology and age in our cross-sectional study suggests a potential association, where younger participants might have had more recent exposure to modern pain education compared to older respondents who may retain more traditional biomedical beliefs. While this association aligns with similar findings from an Italian study [42]—which also reported lower scores among individuals over 40—it is crucial to recognize that a cross-sectional design cannot establish causality. Therefore, we cannot definitively conclude that age causes differences in pain knowledge or belief systems, nor does it confirm that ‘modern education’ is the direct reason for higher scores in younger professionals. This correlation merely highlights an area for further investigation into the temporal and educational factors that might contribute to these observed differences, especially given that other research has reported no age-related differences in similar contexts [40].

As for the HC-PAIRS, the scores vary between 23 and 92 points out of a possible 105, reflecting diversity in alignment with the biopsychosocial model or the biomedical one. The mean average score was 64.97. A systematic review that included 51 studies reported an overall baseline mean score of 55.34 (95% CI: 53.54–57.14). Nurses had the highest scores, whereas physical therapists, physicians, and exercise professionals had lower scores. Compared with the international results, the beliefs of our sample lean slightly towards the more traditional, biomedical approach, which indicates the need for educational programs that promote the biopsychosocial approach [43].

Even though our study did not obtain conclusive results about the connection of NPQ and HC-PAIRS scores, research shows that there can be a negative correlation between the level of pain knowledge and beliefs aligned with the biopsychosocial model [44].

After educational interventions, most studies reported lower scores on the HC-PAIRS [45,46], for example, a study conducted on physical therapy students, who (after the pain education intervention) obtained improved scores, suggesting that these interventions are potentially valuable part of the education of physiotherapy students [47]. On the other hand, one study concluded that after a 12-week pain education module, paradoxically, osteopathy students scored higher on the HC-PAIRS [48].

On the TSK-11 the average score of the respondents was 22, indicating a moderate to insignificant kinesiophobia, with the lowest score being 11, and only one person scoring above 33 points, obtaining results that suggest significant kinesiophobia. Other studies report a much higher prevalence of high kinesiophobia in patients with CLBP [49]. This low prevalence of severe kinesiophobia might reflect specific characteristics of the sample, for example: LBP severity, higher educational attainment (and particularly the attainment of medical education) compared to the general public, or psychological support.

Age-related findings of our study are supported by the literature, suggesting that younger individuals tend to have lower level of kinesiophobia due to greater psychological and physical adaptability, whereas older adults may develop fear of movement as a result of cumulative pain experiences [50].

The significant negative correlation between rNPQ and TSK-11 scores indicate that a higher level knowledge of pain neurophysiology leads to lower level of kinesiophobia. Education plays a key role in treating the fear of movement, as understanding the mechanisms of pain can decrease the perceived threat associated with movement, thereby positively influencing avoidance behavior. Research on this topic is very limited; further investigation is needed to draw a strong conclusion.

A significant negative correlation was identified between rNPQ and TSK-11 scores, suggesting an association where higher reported levels of knowledge of pain neurophysiology tend to coincide with a lower level of kinesiophobia. However, it is imperative to emphasize that, given the cross-sectional nature of this study, we cannot infer a causal relationship from this correlation. While it is often posited that education plays a key role in treating fear of movement—potentially by decreasing perceived threat and influencing avoidant behavior—our data only indicate co-occurrence and do not establish that increased knowledge directly leads to reduced kinesiophobia in this context. The limited existing research on this specific correlation in cross-sectional designs underscore the necessity for future rigorous, preferably longitudinal or interventional, investigation to robustly explore all causal pathways.

## 5. Conclusions

This study highlights gaps in knowledge of pain neurophysiology, and more biomedically oriented beliefs regarding CLBP among healthcare professionals, predominantly physiotherapists.

This study critically examined healthcare professionals’ knowledge of pain neurophysiology, their beliefs about chronic low back pain (CLBP), and the levels of kinesiophobia, primarily among physiotherapists. The main scientific contribution is the clear demonstration of a prevailing moderate-level understanding of pain neurophysiology and a tendency towards biomedical, as opposed to biopsychosocial, beliefs within this group, alongside the crucial finding that greater knowledge of pain neurophysiology is significantly associated with reduced kinesiophobia.

These findings accentuate the need for structured educational interventions and integration of the biopsychosocial model into both university curricula and continuing professional development. Interdisciplinary training and practical modules may enhance understanding, reduce maladaptive fear-avoidance behaviors, and optimize chronic pain management. Future research should involve larger, more diverse samples and evaluate long-term impacts of education on professional practice.

### 5.1. Clinical and Educational Implications

These findings underscore the need to integrate pain neurophysiology education into undergraduate and postgraduate medical curricula. Targeted interventions can reduce dysfunctional beliefs, promote evidence-based chronic pain management, and consolidate learning through practical, on-site modules. Interdisciplinary collaboration is recommended to standardize patient education, enhance understanding of neurophysiological and psychological pain mechanisms, and optimize rehabilitation outcomes.

### 5.2. Study Limitation

The present study has several limitations that may compromise the generalization of the results. First, the sample size is relatively small, which limits the statistical power of the analysis. In particular, the gender imbalance and the small size of the control group reduce the comparative validity between subgroups.

Another limitation is the low representativeness outside the field of physical therapy, which significantly reduces the possibility of interpreting this study’s results as a broad interdisciplinary perspective on the pain knowledge and beliefs of all healthcare specialists involved in rehabilitation. Moreover, the lack of detailed information regarding the participants’ level of professional experience, practice context, and professional interest represents another potentially important factor that may have an impact on pain-related beliefs and was not controlled for this study.

The online questionnaire, although cost-effective and practical, may introduce a selection bias favoring participants with interest in the subject and access to technology. In addition, data collected through self-reporting may lead to interpretation errors. Furthermore, compared to this cross-sectional study, a longitudinal study could provide more valuable information on the impact of education, clinical exposure, or other formative interventions on the evolution of healthcare workers beliefs about chronic pain.

For future studies, we recommend expanding and diversifying the sample, and to integrate interviews into the data collection. Cultural adaptation of the instruments used would facilitate international comparisons and highlight specific cultural influences, such as traditional perceptions of pain.

## Figures and Tables

**Table 1:** Distribution of the participants based on different variables.

Variable	No. of Participants
Gender	
Male	14
Female	28
Age	
Under 25	17
25–30	11
31–40	11
Over 40	3
Occupation	
Physical therapist	16
Physical therapist with physiotherapy modalities	8
Doctor	3
Other (nurse, dietician, etc.)	15
Professional experience (years)	
Under 2	14
2–5	17
6–10	6
Over 10	11
Presence of CLBP	
Yes	34
No	8

**Table 2:** Descriptive statistics for the rNPQ.

rNPQ	Age				Total
Level	<25	25–30	31–40	>40	No.
Low	3	1	5	2	11
Moderate	12	9	6	1	28
High	2	1	0	0	3
Total (No.)	17	11	11	3	42

rNPO = Revised Neurophysiology of Pain Questionnaire; No. = number of participants.

**Table 3:** Distribution of the HC-PAIRS scores.

	Gender		Total	Age				Total
	Female	Male		<25	25–30	31–40	>40	
HC-PAIRS score < 60	9	3	12	2	3	5	2	12
>60	19	11	30	15	8	6	1	30
Total (No.)	28	14	42	17	11	11	3	42

**Table 4:** Statistics regarding HC-PAIRS and gender.

	Levene’s Test for Equality of Variances		*t*-Test for Equality of Means						
	F	Sig.	t	df	Sig. (Two-Tailed)	Mean Difference	Std. Error Difference	95% Confidence Interval of the Difference	
								Lower	Upper
Equal variances assumed	0.089	0.767	−1.265	40	0.213	−6.357	5.027	−16.51	3.802
Equal variances not assumed			−1.234	24.50	0.229	−6.357	5.150	−16.97	4.260

**Table 5:** Descriptive analysis of the TSK-11 scores based on gender and age.

Total Score TSK	Gender		Total	Age				Total
	Female	Male		<25	25–30	31–40	>40	
11–22	16	8	24	13	6	4	1	24
23–33	12	5	17	4	4	7	2	17
>34	0	1	1	0	1	0	0	1
Total (No.)	28	14	42	17	11	11	3	42

TSK-11 = Tampa Scale of Kinesiophobia; No. = number of participants.

**Table 6:** Correlation between rNPQ, TSK-11, HC-PAIRS, and age.

		rNPQ	HCPAIRS	TSK	Age
rNPQ	Pearson Correlation	1	−0.101	−0.389 *	−0.358 *
	Sig. (Two-Tailed)		0.526	0.011	0.020
	N		42	42	42
HC-PAIRS	Pearson Correlation		1	0.214	−0.241
	Sig. (Two-Tailed)			0.173	0.124
	N			42	42
TSK	Pearson Correlation			1	0.432 *
	Sig. (Two-Tailed)				0.004
	N				42

* Correlation is significant at the 0.05 level (two-tailed).

## Data Availability

The original contributions presented in this study are included in the article. Further inquiries can be directed to the corresponding author.
